# The methanol sesquisolvate of sodium naproxen

**DOI:** 10.1107/S2056989018014652

**Published:** 2018-10-19

**Authors:** Helene Kriegner, Matthias Weil, Matthew J. Jones

**Affiliations:** a Institute for Chemical Technologies and Analytics, Division of Structural Chemistry, TU Wien, Getreidemarkt 9/164-SC, A-1060 Vienna, Austria; b Patheon Austria GmbH & Co KG, St.-Peter-Strasse 25, A-4020 Linz, Austria

**Keywords:** crystal structure, solvatomorphism, naproxen, methanol solvate, disorder

## Abstract

The asymmetric unit of the methanol solvate of sodium naproxen, systematic name: sodium (2*S*)-2-(6-meth­oxy­naphthalen-2-yl)propano­ate methanol sesquisolvate, Na^+^·C_14_H_13_O_3_^−^·1.5CH_3_OH, comprises two formula units of the mol­ecular salt and three methanol mol­ecules. One of the sodium cations exhibits a coordination number of six and is bonded to three carboxyl­ate O atoms and three methanol OH groups whereas the second sodium cation has a coordination number of seven, defined by five carboxyl­ate O atoms and two methanol OH groups. Both coordination polyhedra around the sodium cations are considerably distorted. The two types of cations are bridged into polymeric chains extending parallel to [010]. This arrangement is stabilized by intrachain O—H⋯O hydrogen bonds between methanol ligands as donor and carboxyl­ate O atoms as acceptor groups. The hydro­phobic 6-meth­oxy­naphthyl moieties flank the hydro­philic sodium oxygen chains into ribbons parallel to [010]. There are no noticeable inter­molecular inter­actions between these ribbons. One of the 6-meth­oxy­naphthyl moieties is disordered over two sets of sites in a 0.723 (3):0.277 (3) ratio.

## Abstract 2

A new solvatomorph of sodium naproxen with methanol as solvent is reported. The asymmetric unit comprises two formula units of sodium naproxen and three methanol mol­ecules.

## Chemical context   

Naproxen, or (*S*)-2-(6-meth­oxy­naphthalen-2-yl)propanoic acid, and in particular its better soluble sodium salt are non-steroidal anti-inflammatory drugs with pain-relieving and anti­pyretic properties. For a recent project on the crystallization of active pharmaceutical ingredients (APIs; Kovačič *et al.*, 2012 ▸), we used sodium naproxen as a model substance. During these investigations, we obtained the methanol sesquisolvate as a solvatomorph of sodium naproxen, [Na(C_14_H_13_O_3_)]·1.5CH_3_OH. Although a preliminary structure model of this compound has been reported as part of a PhD thesis (Chavez, 2009 ▸), it was never published or deposited in the Cambridge Structural Database (Groom *et al.*, 2016 ▸). We report here the precise crystal structure determination of [Na(C_14_H_13_O_3_)]·1.5CH_3_OH, (I), including disorder of one 6-meth­oxy­naphthyl moiety that was not modelled in the preliminary study (Chavez, 2009 ▸).
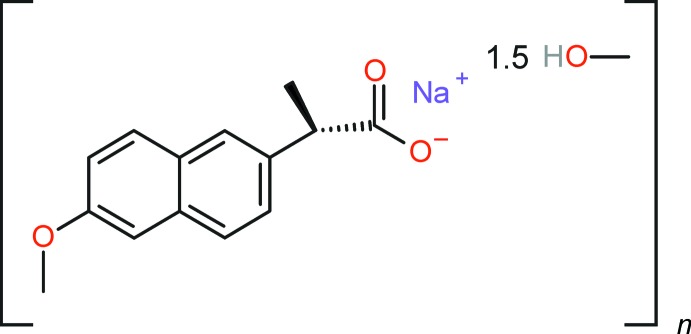


## Structural commentary   

**Figure 1:**
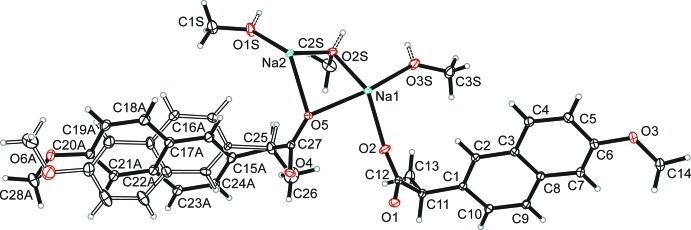
The asymmetric unit of (I) with displacement ellipsoids drawn at the 30% probability level. The minor disordered part *B* of one 6-meth­oxy­naphthyl moiety is displayed with open bonds and without labelling of atoms.

**Figure 2:**
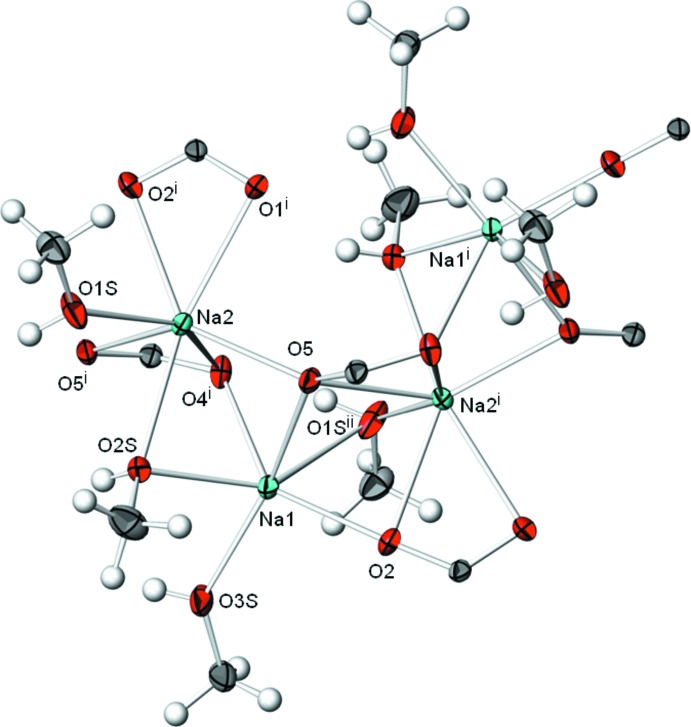
Part of the crystal structure of (I) emphasizing the coordination environments of the two Na^+^ cations. Displacement ellipsoids are drawn at the 50% probability level. Symmetry codes refer to Table 1 ▸.

The asymmetric unit of (I) is displayed in Fig. 1 ▸ and comprises two Na^+^ cations, two naproxate anions (one of which shows disorder of the 6-meth­oxy­naphthyl moiety) and three methanol mol­ecules (*Z*′ = 2). Na1 is bound to six oxygen atoms, three of them originating from methanol OH groups and three from monodentate carboxyl­ate groups (O2; O5; O4^i^; for symmetry codes, see: Table 1 ▸). The Na1—O bond lengths are not uniformly distributed, revealing a distorted [5 + 1] coordination with five shorter bonds between 2.2355 (14) and 2.4403 (14) Å and one significantly longer bond of 2.856 (2) Å to the OH group of a methanol mol­ecule. In comparison, the coordination sphere of Na2 is enlarged to seven coordination partners, two of them from methanol OH groups, four from two chelating carboxyl­ate groups (O1^i^,O2^i^; O4^i^,O5^i^) and one from a monodentate carboxyl­ate group (O5). The Na2—O distances are somewhat more evenly distributed and range from 2.3418 (13) to 2.5983 (14) Å. Nevertheless, the resulting coordination polyhedron around Na2 is likewise distorted. Details of the Na—O coordination spheres are depicted in Fig. 2 ▸. The bond-valence sums (Brown, 2002 ▸) of 1.24 and 1.17 valence units for Na1 and Na2, respectively, are higher than expected and point to some strain in the structure.

**Figure 3:**
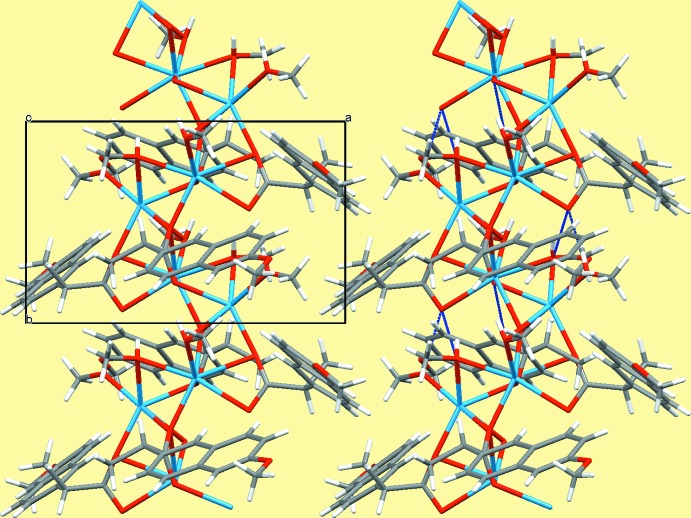
The crystal structure of (I) in a projection along [001]. O—H⋯O hydrogen bonds within a ribbon are displayed in blue on the right hand side. For clarity, only the major part *A* of the disordered 6-meth­oxy­naphthyl moiety is shown.

The two sodium cations are bridged by the O1*S* and O2*S* methanol OH groups and by carboxyl­ate atoms O2, O4 and O5 into zigzag chains extending parallel to [010]. The third methanol mol­ecule is terminally bound to Na1. The hydro­phobic 6-meth­oxy­naphthyl moieties flank the hydro­philic [Na—O]_*n*_ chains, leading to the formation of ribbons along the chain direction (Fig. 3 ▸). The meth­oxy groups attached to the naphthyl rings are twisted slightly out of the aromatic plane, with dihedral angles of 6.42 (18)° for ring (C1–C10) and meth­oxy group O3–C14, and 5.2 (3)° for ring (C15*A*–C24*A*) and meth­oxy group O6*A*–C28*A*.

## Supra­molecular features   

Intra­chain O—H⋯O hydrogen bonding inter­actions of medium strength [Table 2 ▸, Fig. 3 ▸(right)] between methanol mol­ecules and carboxyl­ate O atoms stabilize the arrangement within the ribbons. Oxygen atom O1, which is not a bridging atom in the [Na—O]_*n*_ chain and which has a comparatively long Na—O bond, is the acceptor of two hydrogen bonds.

There are no notable inter­molecular inter­actions between adjacent ribbons involving the outer hydro­phobic parts. It seems that cohesion of the ribbons is dominated by van der Waals forces only.

## Database survey   

The crystal structure of naproxen, *i.e.* the free acid (±)2-(6-meth­oxy-2-naphth­yl)propionic acid, was reported by Ravikumar *et al.* (1985 ▸). A search in the Cambridge Structural Database (CSD version 5.39, November 2017, update 3, May 2018; Groom *et al.*, 2016 ▸) for the sodium salt and its hydrates revealed six entries: anhydrous sodium naproxen, [Na(C_14_H_13_O_3_)] (Kim *et al.*, 2004 ▸), sodium naproxen monohydrate [Na(C_14_H_13_O_3_)]·H_2_O (Kim *et al.*, 1990 ▸), two forms of sodium naproxen dihydrate [Na(C_14_H_13_O_3_)]·2H_2_O (Bond *et al.*, 2014 ▸), and sodium naproxen heminona­hydrate [Na(C_14_H_13_O_3_)]·4.5H_2_O (Burgess *et al.*, 2012 ▸) that was subsequently re­inter­preted as a disordered tetra­hydrate [Na(C_14_H_13_O_3_)]·4H_2_O (Bond *et al.*, 2013 ▸). The structural motif of ribbons formed between sodium cations and oxygen atoms is likewise found in all anhydrous and hydrous sodium naproxen structures.

Only one methanol solvate of naproxen is deposited in the CSD. However, this is an Na salt of naproxen with an additional free acid mol­ecule, *viz.* sodium hydrogen bis­(naproxate) methanol disolvate, [Na(C_14_H_13_O_3_)(C_14_H_14_O_3_)]·2CH_3_OH (Perumalla & Sun, 2012 ▸). A homologous series of alcohol solvates of sodium naproxen obtained as polycrystalline powders and without structure determinations was reported by Chavez *et al.* (2010 ▸). During these investigations, another methanol solvate of sodium naproxen was reported with only one methanol mol­ecule per formula unit (Chavez, 2009 ▸; Burgess *et al.*, 2012 ▸).

## Synthesis and crystallization   

Crystals of sodium naproxen methanol sesquisolvate were grown by slow crystallization in methanol. Polycrystalline anhydrous sodium naproxen was dissolved in methanol to yield a solution 20% in weight of the salt. 0.5 ml of this solution were heated to 338 K and slowly cooled down to room temperature (298 K) over the course of 130 min (cooling rate 0.3 K min^−1^). Colourless parallelepipeds with edge lengths of up to 1 cm were obtained. A suitable fragment was broken from a larger specimen for the X-ray diffraction experiment.

## Refinement details   

Crystal data, data collection and structure refinement details are summarized in Table 3 ▸. The structure model obtained with *SHELXT* (Sheldrick, 2015*a* ▸) was very similar to the preliminary model of Chavez (2009 ▸) from 173 K data using Cu *K*_α_ radiation. After placing all atoms with full occupancy in the asymmetric unit, elongated displacement parameters of atoms of one of the 6-meth­oxy­naphthyl moieties and conspicuous electron density peaks in the vicinity of these atoms were found. This model converged with *R*[*F* ^2^ > 2*σ*(*F* ^2^)] = 0.08 and *wR*(*F* ^2^) = 0.23. Consideration of disorder over two sets of sites for this fragment led to more spherical atoms and much better reliability factors (Table 3 ▸). The refined occupancy ratio of the two disordered parts is 0.723 (3):0.277 (3) for major part *A*: minor part *B*. The positions of C-bound H atoms were calculated and refined using a riding model, with C—H = 0.93–0.98 Å, and with *U*_iso_(H) = 1.2*U*_eq_(C) or 1.5*U*_eq_(C) for methyl H atoms. H atoms bound to methanol O atoms were clearly discernible from difference maps. They were refined with distance restraints of 0.85±2 Å and free *U*_iso_(H) values. The absolute structure was determined on the basis of the current data set (Table 3 ▸), revealing that the usual (*S*) enanti­omer is present.

Reflections (100) and (001) were obstructed by the beam stop and were omitted from the refinement.

Lattice parameters refined from single-crystal room temperature X-ray data are *a* = 12.8458 (9), *b* = 8.0235 (6), *c* = 15.3012 (11) Å, *β* = 94.898 (2)°.

## Supplementary Material

Crystal structure: contains datablock(s) I, global. DOI: 10.1107/S2056989018014652/rz5244sup1.cif

Structure factors: contains datablock(s) I. DOI: 10.1107/S2056989018014652/rz5244Isup2.hkl

CCDC reference: 1873620

Additional supporting information:  crystallographic information; 3D view; checkCIF report

## Figures and Tables

**Table 1:** Selected bond lengths (Å)

Na1—O2	2.2355 (14)	Na2—O1*S*	2.3667 (15)
Na1—O3*S*	2.3003 (15)	Na2—O4^i^	2.4635 (13)
Na1—O5	2.3604 (13)	Na2—O2*S*	2.4748 (14)
Na1—O2*S*	2.3838 (14)	Na2—O1^i^	2.5394 (14)
Na1—O4^i^	2.4403 (14)	Na2—O2^i^	2.5459 (15)
Na1—O1*S* ^ii^	2.856 (2)	Na2—O5^i^	2.5983 (14)
Na2—O5	2.3418 (13)		

Symmetry codes: (i) 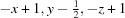; (ii) 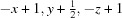.

**Table 2:** Hydrogen-bond geometry (Å, °)

*D*—H⋯*A*	*D*—H	H⋯*A*	*D*⋯*A*	*D*—H⋯*A*
O1*S*—H1*S*⋯O4^iii^	0.84 (2)	1.90 (2)	2.6756 (18)	153 (3)
O2*S*—H2*S*⋯O1^iii^	0.83 (2)	2.06 (2)	2.8331 (18)	156 (3)
O3*S*—H3*S*⋯O1^iii^	0.79 (2)	1.94 (2)	2.7226 (19)	172 (3)

Symmetry code: (iii) 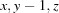.

**Table 3:** Experimental details

Crystal data	
Chemical formula	Na^+^·C_14_H_13_O_3_^−^·1.5CH_3_OH
*M*_r_	300.30
Crystal system, space group	Monoclinic, *P*2_1_
Temperature (K)	100
*a*, *b*, *c* (Å)	12.6776 (9), 7.9675 (6), 15.1932 (11)
β (°)	95.7559 (19)
*V* (Å^3^)	1526.91 (19)
*Z*	4
Radiation type	Mo *K*α
μ (mm^−1^)	0.12
Crystal size (mm)	0.45 × 0.45 × 0.35

Data collection	
Diffractometer	Bruker APEXII CCD
Absorption correction	Multi-scan (*SADABS*; Krause *et al.*, 2015 ▸)
*T*_min_, *T*_max_	0.675, 0.747
No. of measured, independent and observed [*I* > 2σ(*I*)] reflections	32667, 11521, 10262
*R*_int_	0.029
(sin θ/λ)_max_ (Å^−1^)	0.767

Refinement	
*R*[*F* ^2^ > 2σ(*F* ^2^)], *wR*(*F* ^2^), *S*	0.039, 0.105, 1.02
No. of reflections	11521
No. of parameters	508
No. of restraints	4
H-atom treatment	H atoms treated by a mixture of independent and constrained refinement
Δρ_max_, Δρ_min_ (e Å^−3^)	0.43, −0.24
Absolute structure	Flack *x* determined using 4468 quotients [(*I* ^+^)−(*I* ^−^)]/[(*I* ^+^)+(*I* ^−^)] (Parsons *et al.*, 2013 ▸)
Absolute structure parameter	−0.07 (8)

Computer programs: *APEX3* and *SAINT* (Bruker, 2017 ▸), *SHELXT* (Sheldrick, 2015*a* ▸), *SHELXL2017* (Sheldrick, 2015*b* ▸), *Mercury* (Macrae *et al.*, 2008 ▸), *ATOMS* (Dowty, 2006 ▸) and *XP* in *SHELXTL* (Sheldrick, 2008 ▸), *publCIF* (Westrip, 2010 ▸).

## supplementary crystallographic information

### Crystal data

**Table:**

Na^+^·C_14_H_13_O_3_^−^·1.5CH_4_O	*F*(000) = 636
*M**_r_* = 300.30	*D*_x_ = 1.306 Mg m^−^^3^
Monoclinic, *P*2_1_	Mo *K*α radiation, λ = 0.71073 Å
*a* = 12.6776 (9) Å	Cell parameters from 9943 reflections
*b* = 7.9675 (6) Å	θ = 2.5–38.0°
*c* = 15.1932 (11) Å	µ = 0.12 mm^−^^1^
β = 95.7559 (19)°	*T* = 100 K
*V* = 1526.91 (19) Å^3^	Block, colourless
*Z* = 4	0.45 × 0.45 × 0.35 mm

### Data collection

**Table:**

Bruker APEXII CCD diffractometer	10262 reflections with *I* > 2σ(*I*)
ω scans	*R*_int_ = 0.029
Absorption correction: multi-scan (*SADABS*; Krause *et al.*, 2015)	θ_max_ = 33.0°, θ_min_ = 2.2°
*T*_min_ = 0.675, *T*_max_ = 0.747	*h* = −16→19
32667 measured reflections	*k* = −12→12
11521 independent reflections	*l* = −23→22

### Refinement

**Table:**

Refinement on *F*^2^	Hydrogen site location: mixed
Least-squares matrix: full	H atoms treated by a mixture of independent and constrained refinement
*R*[*F*^2^ > 2σ(*F*^2^)] = 0.039	*w* = 1/[σ^2^(*F*_o_^2^) + (0.0585*P*)^2^ + 0.2474*P*] where *P* = (*F*_o_^2^ + 2*F*_c_^2^)/3
*wR*(*F*^2^) = 0.105	(Δ/σ)_max_ = 0.001
*S* = 1.02	Δρ_max_ = 0.43 e Å^−^^3^
11521 reflections	Δρ_min_ = −0.24 e Å^−^^3^
508 parameters	Absolute structure: Flack *x* determined using 4468 quotients [(*I*^+^)-(*I*^-^)]/[(*I*^+^)+(*I*^-^)] (Parsons *et al.*, 2013)
4 restraints	Absolute structure parameter: −0.07 (8)

### Special details

**Table:**

Geometry. All esds (except the esd in the dihedral angle between two l.s. planes) are estimated using the full covariance matrix. The cell esds are taken into account individually in the estimation of esds in distances, angles and torsion angles; correlations between esds in cell parameters are only used when they are defined by crystal symmetry. An approximate (isotropic) treatment of cell esds is used for estimating esds involving l.s. planes.

### Fractional atomic coordinates and isotropic or equivalent isotropic displacement parameters (Å^2^)

**Table:**

	*x*	*y*	*z*	*U*_iso_*/*U*_eq_	Occ. (<1)
Na1	0.35919 (5)	0.41121 (8)	0.55421 (5)	0.01949 (13)	
Na2	0.53085 (5)	0.27034 (8)	0.43935 (4)	0.01651 (12)	
O1	0.30172 (10)	0.93243 (15)	0.58392 (9)	0.0234 (2)	
O2	0.27973 (11)	0.66163 (15)	0.55460 (10)	0.0259 (3)	
O3	0.09118 (12)	0.70972 (19)	1.09131 (8)	0.0282 (3)	
O4	0.46523 (10)	0.79820 (14)	0.39887 (8)	0.0209 (2)	
O5	0.44988 (10)	0.53419 (14)	0.44218 (8)	0.0194 (2)	
C1	0.09928 (11)	0.80698 (17)	0.67179 (9)	0.0141 (2)	
C2	0.16031 (12)	0.71291 (19)	0.73342 (10)	0.0167 (2)	
H2	0.218745	0.651509	0.715525	0.020*	
C3	0.13817 (12)	0.7053 (2)	0.82309 (10)	0.0177 (3)	
C4	0.20276 (15)	0.6122 (2)	0.88774 (11)	0.0245 (3)	
H4	0.259778	0.546767	0.870353	0.029*	
C5	0.18346 (15)	0.6161 (2)	0.97496 (12)	0.0255 (3)	
H5	0.227230	0.553456	1.017487	0.031*	
C6	0.09883 (15)	0.7127 (2)	1.00203 (11)	0.0228 (3)	
C7	0.03229 (13)	0.7995 (2)	0.94124 (10)	0.0205 (3)	
H7	−0.025886	0.860850	0.959611	0.025*	
C8	0.05126 (12)	0.79659 (19)	0.85034 (10)	0.0173 (3)	
C9	−0.01335 (12)	0.8878 (2)	0.78550 (10)	0.0187 (3)	
H9	−0.073439	0.946900	0.801972	0.022*	
C10	0.00991 (12)	0.89198 (19)	0.69887 (10)	0.0171 (3)	
H10	−0.034925	0.953119	0.656440	0.020*	
C11	0.12458 (12)	0.82248 (19)	0.57623 (10)	0.0164 (2)	
H11	0.104366	0.938450	0.555788	0.020*	
C12	0.24397 (12)	0.80296 (18)	0.57028 (10)	0.0161 (2)	
C13	0.05919 (15)	0.6995 (3)	0.51580 (12)	0.0306 (4)	
H13A	0.077854	0.584172	0.533647	0.046*	
H13B	0.074224	0.717156	0.454462	0.046*	
H13C	−0.016427	0.718215	0.520568	0.046*	
C14	0.0128 (2)	0.8144 (3)	1.12363 (13)	0.0371 (5)	
H14A	0.025640	0.931340	1.107814	0.056*	
H14B	0.016201	0.804060	1.188131	0.056*	
H14C	−0.057606	0.779923	1.097220	0.056*	
C27	0.43419 (12)	0.64886 (19)	0.38570 (10)	0.0168 (3)	
C26	0.2643 (2)	0.6973 (4)	0.29275 (16)	0.0477 (6)	
H26A	0.275881	0.818801	0.290702	0.072*	
H26B	0.228868	0.669292	0.345285	0.072*	
H26C	0.219732	0.661993	0.239531	0.072*	
C25	0.37115 (15)	0.6064 (2)	0.29702 (11)	0.0256 (3)	
H25A	0.357320	0.482859	0.295488	0.031*	0.723 (3)
H25B	0.352875	0.484963	0.302090	0.031*	0.277 (3)
C15A	0.4275 (3)	0.6512 (5)	0.2181 (2)	0.0216 (6)	0.723 (3)
C16A	0.5293 (2)	0.5923 (4)	0.21714 (16)	0.0245 (5)	0.723 (3)
H16A	0.561067	0.532294	0.266975	0.029*	0.723 (3)
C17A	0.5886 (2)	0.6187 (3)	0.14351 (18)	0.0227 (5)	0.723 (3)
C18A	0.6929 (2)	0.5558 (4)	0.14182 (16)	0.0274 (5)	0.723 (3)
H18A	0.725846	0.496607	0.191447	0.033*	0.723 (3)
C19A	0.7463 (2)	0.5801 (4)	0.06897 (16)	0.0265 (5)	0.723 (3)
H19A	0.815841	0.535976	0.068427	0.032*	0.723 (3)
C20A	0.6998 (2)	0.6698 (4)	−0.00588 (19)	0.0205 (5)	0.723 (3)
C21A	0.59938 (19)	0.7341 (3)	−0.00630 (14)	0.0218 (5)	0.723 (3)
H21A	0.568545	0.795556	−0.055962	0.026*	0.723 (3)
C22A	0.5413 (3)	0.7079 (5)	0.0688 (2)	0.0212 (6)	0.723 (3)
C23A	0.43633 (19)	0.7676 (3)	0.07052 (14)	0.0231 (5)	0.723 (3)
H23A	0.403419	0.827833	0.021220	0.028*	0.723 (3)
C24A	0.3816 (2)	0.7395 (3)	0.14262 (16)	0.0241 (5)	0.723 (3)
H24A	0.311058	0.780256	0.141984	0.029*	0.723 (3)
C28A	0.7197 (2)	0.7553 (5)	−0.1545 (2)	0.0315 (6)	0.723 (3)
H28A	0.656366	0.692641	−0.177536	0.047*	0.723 (3)
H28B	0.772161	0.751836	−0.197667	0.047*	0.723 (3)
H28C	0.700449	0.872125	−0.143910	0.047*	0.723 (3)
O6A	0.76404 (16)	0.6807 (3)	−0.07288 (12)	0.0237 (4)	0.723 (3)
O1S	0.50266 (14)	0.08627 (18)	0.31699 (10)	0.0341 (3)	
C1S	0.55409 (18)	0.0686 (3)	0.24025 (14)	0.0322 (4)	
H1S1	0.587260	0.175409	0.226716	0.048*	
H1S2	0.502388	0.036587	0.190801	0.048*	
H1S3	0.608648	−0.018496	0.249473	0.048*	
O2S	0.34838 (10)	0.17528 (15)	0.45724 (8)	0.0205 (2)	
C2S	0.25390 (17)	0.1708 (3)	0.39960 (16)	0.0391 (5)	
H2S1	0.195993	0.128143	0.431475	0.059*	
H2S2	0.263523	0.096897	0.349473	0.059*	
H2S3	0.236603	0.284294	0.377843	0.059*	
O3S	0.24271 (15)	0.24954 (18)	0.62435 (13)	0.0412 (4)	
C3S	0.16678 (18)	0.2742 (3)	0.68361 (15)	0.0337 (4)	
H3S1	0.202144	0.285994	0.743672	0.051*	
H3S2	0.118646	0.177752	0.681302	0.051*	
H3S3	0.126265	0.376335	0.667452	0.051*	
O6B	0.7222 (7)	0.7340 (10)	−0.1082 (7)	0.050 (2)	0.277 (3)
C15B	0.4588 (7)	0.6105 (10)	0.2265 (5)	0.0158 (13)	0.277 (3)
C24B	0.5534 (7)	0.5183 (10)	0.2415 (5)	0.0313 (16)	0.277 (3)
H24B	0.566878	0.454601	0.294247	0.038*	0.277 (3)
C23B	0.6268 (7)	0.5193 (10)	0.1806 (6)	0.0365 (18)	0.277 (3)
H23B	0.689893	0.454986	0.191046	0.044*	0.277 (3)
C22B	0.6085 (6)	0.6161 (9)	0.1022 (5)	0.0292 (15)	0.277 (3)
C21B	0.6837 (7)	0.6204 (10)	0.0362 (6)	0.0324 (17)	0.277 (3)
H21B	0.747181	0.556454	0.043704	0.039*	0.277 (3)
C20B	0.6611 (8)	0.7178 (12)	−0.0363 (6)	0.0355 (19)	0.277 (3)
C19B	0.5698 (8)	0.8163 (13)	−0.0498 (5)	0.045 (2)	0.277 (3)
H19B	0.558921	0.887368	−0.100039	0.054*	0.277 (3)
C18B	0.4966 (7)	0.8096 (12)	0.0097 (5)	0.0380 (18)	0.277 (3)
H18B	0.432892	0.872406	−0.001075	0.046*	0.277 (3)
C16B	0.4398 (6)	0.7027 (8)	0.1519 (4)	0.0204 (11)	0.277 (3)
H16B	0.375346	0.763864	0.142336	0.024*	0.277 (3)
C17B	0.5134 (9)	0.7109 (14)	0.0874 (7)	0.027 (2)	0.277 (3)
C28B	0.7973 (8)	0.6136 (14)	−0.1092 (8)	0.049 (2)	0.277 (3)
H28D	0.854049	0.635146	−0.061943	0.073*	0.277 (3)
H28E	0.826577	0.614705	−0.166515	0.073*	0.277 (3)
H28F	0.765540	0.503621	−0.099974	0.073*	0.277 (3)
H3S	0.256 (3)	0.155 (3)	0.615 (2)	0.049 (9)*	
H1S	0.478 (2)	−0.009 (3)	0.3271 (18)	0.033 (7)*	
H2S	0.350 (2)	0.090 (3)	0.4883 (18)	0.039 (8)*	

### Atomic displacement parameters (Å^2^)

**Table:**

	*U*^11^	*U*^22^	*U*^33^	*U*^12^	*U*^13^	*U*^23^
Na1	0.0218 (3)	0.0118 (3)	0.0264 (3)	0.0010 (2)	0.0102 (2)	0.0011 (2)
Na2	0.0174 (3)	0.0133 (3)	0.0193 (3)	0.0021 (2)	0.0045 (2)	−0.0010 (2)
O1	0.0205 (5)	0.0154 (5)	0.0356 (7)	−0.0030 (4)	0.0100 (5)	0.0017 (4)
O2	0.0260 (6)	0.0152 (5)	0.0384 (7)	0.0048 (4)	0.0118 (5)	−0.0042 (5)
O3	0.0387 (7)	0.0308 (6)	0.0159 (5)	0.0053 (6)	0.0077 (5)	0.0033 (5)
O4	0.0310 (6)	0.0131 (4)	0.0182 (5)	−0.0021 (4)	0.0003 (4)	0.0015 (4)
O5	0.0235 (5)	0.0130 (4)	0.0231 (5)	0.0036 (4)	0.0089 (4)	0.0029 (4)
C1	0.0133 (6)	0.0129 (5)	0.0164 (6)	−0.0020 (4)	0.0033 (4)	−0.0023 (5)
C2	0.0167 (6)	0.0164 (6)	0.0173 (6)	0.0027 (5)	0.0033 (5)	−0.0008 (5)
C3	0.0188 (6)	0.0177 (6)	0.0168 (6)	0.0021 (5)	0.0028 (5)	0.0000 (5)
C4	0.0274 (8)	0.0264 (8)	0.0201 (7)	0.0093 (6)	0.0042 (6)	0.0024 (6)
C5	0.0299 (9)	0.0273 (8)	0.0195 (7)	0.0075 (7)	0.0030 (6)	0.0048 (6)
C6	0.0290 (8)	0.0232 (7)	0.0169 (6)	0.0001 (6)	0.0053 (6)	0.0010 (6)
C7	0.0231 (7)	0.0215 (7)	0.0177 (6)	0.0012 (5)	0.0062 (5)	−0.0010 (5)
C8	0.0184 (6)	0.0174 (6)	0.0166 (6)	−0.0002 (5)	0.0042 (5)	−0.0015 (5)
C9	0.0176 (6)	0.0201 (6)	0.0189 (6)	0.0026 (5)	0.0045 (5)	−0.0015 (5)
C10	0.0148 (6)	0.0184 (6)	0.0180 (6)	0.0006 (5)	0.0019 (5)	−0.0010 (5)
C11	0.0154 (6)	0.0184 (6)	0.0156 (6)	0.0014 (5)	0.0028 (5)	−0.0012 (5)
C12	0.0177 (6)	0.0145 (6)	0.0170 (6)	0.0016 (5)	0.0061 (5)	0.0011 (5)
C13	0.0248 (8)	0.0434 (10)	0.0236 (8)	−0.0100 (8)	0.0023 (6)	−0.0124 (7)
C14	0.0542 (13)	0.0394 (11)	0.0200 (8)	0.0117 (10)	0.0146 (8)	0.0036 (7)
C27	0.0203 (7)	0.0154 (6)	0.0156 (6)	0.0000 (5)	0.0053 (5)	−0.0014 (5)
C26	0.0367 (12)	0.0679 (17)	0.0352 (11)	0.0031 (12)	−0.0127 (9)	−0.0064 (11)
C25	0.0326 (9)	0.0282 (8)	0.0162 (7)	−0.0105 (7)	0.0036 (6)	−0.0049 (6)
C15A	0.0222 (16)	0.0255 (15)	0.0171 (11)	0.0012 (11)	0.0022 (10)	−0.0058 (11)
C16A	0.0302 (14)	0.0296 (13)	0.0134 (9)	0.0069 (10)	0.0002 (8)	−0.0005 (9)
C17A	0.0248 (11)	0.0295 (12)	0.0133 (10)	0.0081 (9)	−0.0008 (8)	0.0013 (8)
C18A	0.0286 (12)	0.0351 (13)	0.0178 (10)	0.0134 (10)	−0.0007 (8)	0.0027 (9)
C19A	0.0256 (12)	0.0337 (13)	0.0201 (10)	0.0108 (10)	0.0019 (9)	0.0002 (9)
C20A	0.0186 (11)	0.0251 (11)	0.0181 (11)	0.0053 (9)	0.0029 (9)	0.0005 (9)
C21A	0.0214 (10)	0.0288 (11)	0.0145 (9)	0.0053 (8)	−0.0010 (8)	−0.0008 (8)
C22A	0.0202 (15)	0.0287 (13)	0.0138 (14)	0.0072 (12)	−0.0026 (10)	−0.0001 (11)
C23A	0.0207 (10)	0.0309 (11)	0.0169 (9)	0.0081 (9)	−0.0025 (7)	0.0006 (8)
C24A	0.0232 (12)	0.0299 (12)	0.0187 (9)	0.0040 (10)	−0.0004 (8)	−0.0030 (8)
C28A	0.0321 (14)	0.0450 (16)	0.0169 (10)	−0.0077 (11)	−0.0006 (10)	0.0056 (11)
O6A	0.0191 (8)	0.0346 (10)	0.0178 (8)	0.0078 (7)	0.0040 (6)	0.0033 (7)
O1S	0.0552 (9)	0.0230 (6)	0.0274 (6)	−0.0187 (6)	0.0211 (6)	−0.0076 (5)
C1S	0.0385 (11)	0.0342 (10)	0.0251 (8)	−0.0100 (8)	0.0094 (7)	−0.0034 (7)
O2S	0.0200 (5)	0.0151 (5)	0.0267 (6)	−0.0003 (4)	0.0033 (4)	0.0007 (4)
C2S	0.0284 (10)	0.0434 (12)	0.0436 (11)	−0.0081 (9)	−0.0065 (8)	0.0083 (10)
O3S	0.0528 (10)	0.0150 (6)	0.0630 (11)	−0.0036 (6)	0.0417 (8)	−0.0020 (6)
C3S	0.0402 (10)	0.0241 (8)	0.0405 (10)	−0.0074 (8)	0.0214 (8)	−0.0073 (8)
O6B	0.051 (5)	0.037 (4)	0.064 (6)	−0.009 (3)	0.025 (4)	−0.009 (4)
C15B	0.018 (4)	0.014 (3)	0.016 (3)	0.003 (2)	0.006 (3)	−0.002 (2)
C24B	0.037 (4)	0.030 (3)	0.029 (3)	0.015 (3)	0.012 (3)	0.012 (3)
C23B	0.037 (4)	0.033 (4)	0.043 (4)	0.016 (3)	0.021 (3)	0.009 (3)
C22B	0.045 (4)	0.024 (3)	0.022 (3)	−0.011 (3)	0.017 (3)	−0.009 (2)
C21B	0.039 (4)	0.024 (3)	0.036 (4)	−0.007 (3)	0.015 (3)	−0.009 (3)
C20B	0.044 (5)	0.030 (4)	0.035 (4)	−0.016 (4)	0.016 (4)	−0.015 (3)
C19B	0.056 (5)	0.059 (6)	0.019 (3)	−0.025 (5)	0.001 (3)	0.006 (3)
C18B	0.044 (4)	0.050 (5)	0.020 (3)	−0.008 (4)	−0.001 (3)	0.012 (3)
C16B	0.023 (3)	0.019 (2)	0.018 (2)	0.003 (2)	−0.006 (2)	0.0044 (19)
C17B	0.040 (6)	0.022 (3)	0.021 (4)	−0.009 (4)	0.008 (3)	−0.004 (3)
C28B	0.031 (4)	0.051 (5)	0.063 (6)	−0.006 (4)	−0.002 (4)	0.013 (5)

### Geometric parameters (Å, º)

**Table:**

Na1—O2	2.2355 (14)	C16A—C17A	1.424 (4)
Na1—O3S	2.3003 (15)	C16A—H16A	0.9500
Na1—O5	2.3604 (13)	C17A—C18A	1.417 (4)
Na1—O2S	2.3838 (14)	C17A—C22A	1.420 (4)
Na1—O4^i^	2.4403 (14)	C18A—C19A	1.368 (4)
Na1—O1S^ii^	2.856 (2)	C18A—H18A	0.9500
Na2—O5	2.3418 (13)	C19A—C20A	1.420 (4)
Na2—O1S	2.3667 (15)	C19A—H19A	0.9500
Na2—O4^i^	2.4635 (13)	C20A—O6A	1.368 (3)
Na2—O2S	2.4748 (14)	C20A—C21A	1.372 (3)
Na2—O1^i^	2.5394 (14)	C21A—C22A	1.434 (4)
Na2—O2^i^	2.5459 (15)	C21A—H21A	0.9500
Na2—O5^i^	2.5983 (14)	C22A—C23A	1.416 (4)
O1—C12	1.2700 (19)	C23A—C24A	1.373 (3)
O2—C12	1.2457 (18)	C23A—H23A	0.9500
O3—C6	1.3698 (19)	C24A—H24A	0.9500
O3—C14	1.422 (3)	C28A—O6A	1.437 (3)
O4—C27	1.2629 (18)	C28A—H28A	0.9800
O5—C27	1.2555 (19)	C28A—H28B	0.9800
C1—C2	1.375 (2)	C28A—H28C	0.9800
C1—C10	1.416 (2)	O1S—C1S	1.399 (2)
C1—C11	1.523 (2)	O1S—H1S	0.84 (2)
C2—C3	1.420 (2)	C1S—H1S1	0.9800
C2—H2	0.9500	C1S—H1S2	0.9800
C3—C8	1.416 (2)	C1S—H1S3	0.9800
C3—C4	1.423 (2)	O2S—C2S	1.411 (2)
C4—C5	1.372 (2)	O2S—H2S	0.83 (2)
C4—H4	0.9500	C2S—H2S1	0.9800
C5—C6	1.415 (2)	C2S—H2S2	0.9800
C5—H5	0.9500	C2S—H2S3	0.9800
C6—C7	1.373 (2)	O3S—C3S	1.396 (2)
C7—C8	1.426 (2)	O3S—H3S	0.79 (2)
C7—H7	0.9500	C3S—H3S1	0.9800
C8—C9	1.417 (2)	C3S—H3S2	0.9800
C9—C10	1.378 (2)	C3S—H3S3	0.9800
C9—H9	0.9500	O6B—C28B	1.352 (13)
C10—H10	0.9500	O6B—C20B	1.408 (11)
C11—C13	1.529 (2)	C15B—C16B	1.352 (10)
C11—C12	1.533 (2)	C15B—C24B	1.405 (11)
C11—H11	1.0000	C24B—C23B	1.376 (10)
C13—H13A	0.9800	C24B—H24B	0.9500
C13—H13B	0.9800	C23B—C22B	1.417 (11)
C13—H13C	0.9800	C23B—H23B	0.9500
C14—H14A	0.9800	C22B—C17B	1.421 (15)
C14—H14B	0.9800	C22B—C21B	1.452 (11)
C14—H14C	0.9800	C21B—C20B	1.355 (14)
C27—C25	1.534 (2)	C21B—H21B	0.9500
C26—C25	1.532 (3)	C20B—C19B	1.396 (16)
C26—H26A	0.9800	C19B—C18B	1.359 (12)
C26—H26B	0.9800	C19B—H19B	0.9500
C26—H26C	0.9800	C18B—C17B	1.417 (13)
C25—C15A	1.499 (4)	C18B—H18B	0.9500
C25—C15B	1.620 (8)	C16B—C17B	1.421 (11)
C25—H25A	1.0000	C16B—H16B	0.9500
C25—H25B	1.0000	C28B—H28D	0.9800
C15A—C16A	1.375 (4)	C28B—H28E	0.9800
C15A—C24A	1.420 (4)	C28B—H28F	0.9800
			
O2—Na1—O3S	100.84 (6)	C26—C25—C15B	128.7 (3)
O2—Na1—O5	83.32 (5)	C27—C25—C15B	104.2 (3)
O3S—Na1—O5	161.49 (7)	C15A—C25—H25A	108.2
O2—Na1—O2S	135.03 (6)	C26—C25—H25A	108.2
O3S—Na1—O2S	81.08 (5)	C27—C25—H25A	108.2
O5—Na1—O2S	83.32 (5)	C26—C25—H25B	104.5
O2—Na1—O4^i^	136.61 (6)	C27—C25—H25B	104.5
O3S—Na1—O4^i^	105.50 (6)	C15B—C25—H25B	104.5
O5—Na1—O4^i^	82.41 (5)	C16A—C15A—C24A	118.0 (3)
O2S—Na1—O4^i^	83.33 (4)	C16A—C15A—C25	116.8 (3)
O2—Na1—O1S^ii^	78.92 (5)	C24A—C15A—C25	125.0 (3)
O3S—Na1—O1S^ii^	109.52 (6)	C15A—C16A—C17A	121.9 (3)
O5—Na1—O1S^ii^	88.96 (5)	C15A—C16A—H16A	119.1
O2S—Na1—O1S^ii^	143.32 (5)	C17A—C16A—H16A	119.1
O4^i^—Na1—O1S^ii^	60.08 (4)	C18A—C17A—C22A	118.8 (3)
O5—Na2—O1S	122.60 (6)	C18A—C17A—C16A	121.9 (2)
O5—Na2—O4^i^	82.29 (4)	C22A—C17A—C16A	119.2 (3)
O1S—Na2—O4^i^	145.68 (5)	C19A—C18A—C17A	120.2 (2)
O5—Na2—O2S	81.75 (4)	C19A—C18A—H18A	119.9
O1S—Na2—O2S	80.06 (5)	C17A—C18A—H18A	119.9
O4^i^—Na2—O2S	81.00 (5)	C18A—C19A—C20A	121.3 (2)
O5—Na2—O1^i^	85.41 (4)	C18A—C19A—H19A	119.3
O1S—Na2—O1^i^	105.56 (5)	C20A—C19A—H19A	119.3
O4^i^—Na2—O1^i^	99.18 (5)	O6A—C20A—C21A	126.4 (3)
O2S—Na2—O1^i^	167.02 (5)	O6A—C20A—C19A	113.4 (2)
O5—Na2—O2^i^	135.92 (5)	C21A—C20A—C19A	120.1 (2)
O1S—Na2—O2^i^	83.41 (6)	C20A—C21A—C22A	119.5 (3)
O4^i^—Na2—O2^i^	94.01 (5)	C20A—C21A—H21A	120.3
O2S—Na2—O2^i^	141.32 (5)	C22A—C21A—H21A	120.3
O1^i^—Na2—O2^i^	51.64 (4)	C23A—C22A—C17A	118.4 (3)
O5—Na2—O5^i^	130.41 (3)	C23A—C22A—C21A	121.6 (3)
O1S—Na2—O5^i^	95.26 (5)	C17A—C22A—C21A	120.0 (3)
O4^i^—Na2—O5^i^	51.90 (4)	C24A—C23A—C22A	120.8 (2)
O2S—Na2—O5^i^	74.12 (4)	C24A—C23A—H23A	119.6
O1^i^—Na2—O5^i^	116.29 (5)	C22A—C23A—H23A	119.6
O2^i^—Na2—O5^i^	72.89 (4)	C23A—C24A—C15A	121.7 (3)
C12—O1—Na2^ii^	92.32 (9)	C23A—C24A—H24A	119.1
C12—O2—Na1	168.53 (13)	C15A—C24A—H24A	119.1
C12—O2—Na2^ii^	92.62 (10)	O6A—C28A—H28A	109.5
Na1—O2—Na2^ii^	83.11 (5)	O6A—C28A—H28B	109.5
C6—O3—C14	116.94 (15)	H28A—C28A—H28B	109.5
C27—O4—Na1^ii^	130.88 (11)	O6A—C28A—H28C	109.5
C27—O4—Na2^ii^	92.75 (9)	H28A—C28A—H28C	109.5
Na1^ii^—O4—Na2^ii^	79.35 (4)	H28B—C28A—H28C	109.5
C27—O5—Na2	132.87 (10)	C20A—O6A—C28A	117.2 (2)
C27—O5—Na1	137.71 (10)	C1S—O1S—Na2	132.12 (12)
Na2—O5—Na1	83.49 (4)	C1S—O1S—Na1^i^	102.53 (14)
C27—O5—Na2^ii^	86.79 (9)	Na2—O1S—Na1^i^	74.36 (5)
Na2—O5—Na2^ii^	130.68 (5)	C1S—O1S—H1S	105.9 (19)
Na1—O5—Na2^ii^	79.62 (4)	Na2—O1S—H1S	116.5 (19)
C2—C1—C10	118.34 (13)	Na1^i^—O1S—H1S	69.3 (19)
C2—C1—C11	122.36 (13)	O1S—C1S—H1S1	109.5
C10—C1—C11	119.30 (13)	O1S—C1S—H1S2	109.5
C1—C2—C3	121.66 (13)	H1S1—C1S—H1S2	109.5
C1—C2—H2	119.2	O1S—C1S—H1S3	109.5
C3—C2—H2	119.2	H1S1—C1S—H1S3	109.5
C8—C3—C2	119.54 (14)	H1S2—C1S—H1S3	109.5
C8—C3—C4	118.47 (14)	C2S—O2S—Na1	113.32 (13)
C2—C3—C4	121.97 (14)	C2S—O2S—Na2	133.15 (13)
C5—C4—C3	120.60 (15)	Na1—O2S—Na2	80.22 (4)
C5—C4—H4	119.7	C2S—O2S—H2S	107 (2)
C3—C4—H4	119.7	Na1—O2S—H2S	108 (2)
C4—C5—C6	120.47 (16)	Na2—O2S—H2S	110 (2)
C4—C5—H5	119.8	O2S—C2S—H2S1	109.5
C6—C5—H5	119.8	O2S—C2S—H2S2	109.5
O3—C6—C7	125.10 (16)	H2S1—C2S—H2S2	109.5
O3—C6—C5	114.18 (15)	O2S—C2S—H2S3	109.5
C7—C6—C5	120.73 (15)	H2S1—C2S—H2S3	109.5
C6—C7—C8	119.44 (15)	H2S2—C2S—H2S3	109.5
C6—C7—H7	120.3	C3S—O3S—Na1	137.38 (12)
C8—C7—H7	120.3	C3S—O3S—H3S	115 (2)
C3—C8—C9	118.21 (13)	Na1—O3S—H3S	108 (2)
C3—C8—C7	120.21 (14)	O3S—C3S—H3S1	109.5
C9—C8—C7	121.56 (14)	O3S—C3S—H3S2	109.5
C10—C9—C8	120.81 (14)	H3S1—C3S—H3S2	109.5
C10—C9—H9	119.6	O3S—C3S—H3S3	109.5
C8—C9—H9	119.6	H3S1—C3S—H3S3	109.5
C9—C10—C1	121.35 (14)	H3S2—C3S—H3S3	109.5
C9—C10—H10	119.3	C28B—O6B—C20B	112.7 (10)
C1—C10—H10	119.3	C16B—C15B—C24B	119.8 (7)
C1—C11—C13	111.54 (13)	C16B—C15B—C25	119.3 (6)
C1—C11—C12	110.58 (12)	C24B—C15B—C25	120.9 (6)
C13—C11—C12	112.24 (13)	C23B—C24B—C15B	120.8 (7)
C1—C11—H11	107.4	C23B—C24B—H24B	119.6
C13—C11—H11	107.4	C15B—C24B—H24B	119.6
C12—C11—H11	107.4	C24B—C23B—C22B	120.2 (7)
O2—C12—O1	123.40 (14)	C24B—C23B—H23B	119.9
O2—C12—C11	118.90 (14)	C22B—C23B—H23B	119.9
O1—C12—C11	117.67 (13)	C23B—C22B—C17B	119.1 (7)
O2—C12—Na2^ii^	61.83 (9)	C23B—C22B—C21B	122.0 (8)
O1—C12—Na2^ii^	61.59 (8)	C17B—C22B—C21B	118.9 (8)
C11—C12—Na2^ii^	179.19 (11)	C20B—C21B—C22B	118.4 (8)
C11—C13—H13A	109.5	C20B—C21B—H21B	120.8
C11—C13—H13B	109.5	C22B—C21B—H21B	120.8
H13A—C13—H13B	109.5	C21B—C20B—C19B	123.1 (7)
C11—C13—H13C	109.5	C21B—C20B—O6B	126.6 (10)
H13A—C13—H13C	109.5	C19B—C20B—O6B	110.4 (9)
H13B—C13—H13C	109.5	C18B—C19B—C20B	119.5 (8)
O3—C14—H14A	109.5	C18B—C19B—H19B	120.3
O3—C14—H14B	109.5	C20B—C19B—H19B	120.3
H14A—C14—H14B	109.5	C19B—C18B—C17B	121.3 (10)
O3—C14—H14C	109.5	C19B—C18B—H18B	119.3
H14A—C14—H14C	109.5	C17B—C18B—H18B	119.3
H14B—C14—H14C	109.5	C15B—C16B—C17B	121.9 (8)
O5—C27—O4	123.49 (14)	C15B—C16B—H16B	119.0
O5—C27—C25	118.22 (14)	C17B—C16B—H16B	119.0
O4—C27—C25	118.26 (14)	C18B—C17B—C16B	123.1 (10)
C25—C26—H26A	109.5	C18B—C17B—C22B	118.8 (9)
C25—C26—H26B	109.5	C16B—C17B—C22B	118.1 (9)
H26A—C26—H26B	109.5	O6B—C28B—H28D	109.5
C25—C26—H26C	109.5	O6B—C28B—H28E	109.5
H26A—C26—H26C	109.5	H28D—C28B—H28E	109.5
H26B—C26—H26C	109.5	O6B—C28B—H28F	109.5
C15A—C25—C26	110.1 (2)	H28D—C28B—H28F	109.5
C15A—C25—C27	113.69 (18)	H28E—C28B—H28F	109.5
C26—C25—C27	108.29 (15)		

Symmetry codes: (i) −*x*+1, *y*−1/2, −*z*+1; (ii) −*x*+1, *y*+1/2, −*z*+1.

### Hydrogen-bond geometry (Å, º)

**Table:**

*D*—H···*A*	*D*—H	H···*A*	*D*···*A*	*D*—H···*A*
O1*S*—H1*S*···O4^iii^	0.84 (2)	1.90 (2)	2.6756 (18)	153 (3)
O2*S*—H2*S*···O1^iii^	0.83 (2)	2.06 (2)	2.8331 (18)	156 (3)
O3*S*—H3*S*···O1^iii^	0.79 (2)	1.94 (2)	2.7226 (19)	172 (3)

Symmetry code: (iii) *x*, *y*−1, *z*.
